# Investigating Association of rs5918 Human Platelets Antigen 1 and rs1800790 Fibrinogen β Chain as Critical Players with Recurrent Pregnancy Loss

**DOI:** 10.3390/medsci6040098

**Published:** 2018-10-31

**Authors:** Fatemeh Karami, Maliheh Askari, Mohammad Hossein Modarressi

**Affiliations:** 1 Department of Medical Genetics, Applied Biophotonics Research Center, Science and Research Branch, Islamic Azad University, Tehran 1477893855, Iran; fateme.karami@gmail.com; 2 Department of Biology, School of Basic Sciences, Science and Research Branch, Islamic Azad University, Tehran 1477893855, Iran; askari.orchid20@gmail.com; 3 Department of Medical Genetics, School of Medicine, Tehran University of Medical Sciences, Tehran 1417653761, Iran

**Keywords:** rs5918, rs1800790, recurrent pregnancy loss, gene polymorphism

## Abstract

Thrombophilia gene variants have been shown to be associated with higher risk of recurrent pregnancy loss (RPL). Due to the role of human platelets antigen 1 (*HPA-1*) and fibrinogen β chain (FGB) as critical players in the coagulation process, their most important variants including rs5918 T > C and rs1800790 G > A were selected to be studied in women affected by RPL. Three milliliters of peripheral blood were drawn from 110 women with history of at least two consecutive spontaneous abortion and 110 healthy women controls. rs5918 T > C and rs1800790 G > A of *HPA-1* and *FGB* genes, respectively, were selected to be analyzed through polymerase chain reaction-restriction fragment length polymorphism (PCR_RFLP) following DNA isolation using QIAamp DNA Blood Mini Kit. Heterozygote genotype (TC) of *HPA-1* gene rs5918 polymorphism was significantly associated with risk of RPL (*p*-value = 0.02). Although, rs1800790 G > A of *FGB* gene was not associated with RPL, its combination with rs5918 polymorphism was associated with increased risk of RPL. Owing to the critical roles of *FGB* and *HPA-1* genes in coagulation, and thrombosis and several confinements on the meaningful association between the combination of those polymorphism with risk of RPL, including them in the thrombophilia panel may increase detection rate of hereditary thrombophilia patients. However, further studies with larger sample sizes are required to shed light on the exact role of the studied gene polymorphism, especially rs1800790 G > A of *FGB* gene variant in pathogenesis of RPL.

## 1. Introduction

Recurrent abortion, which is also known as recurrent pregnancy loss (RPL), is defined as the occurrence of two or more consecutive spontaneous abortions before the 22nd week of gestation. RPL has a general frequency of about 5% among all pregnancies (and, 1% of all couples). Although there is almost no clinically apparent fetal or maternal disturbances, vascular accidents in the placenta can be considered as the main etiology behind unknown cases of repeated abortions [1,2]. Genetic alterations in all the genes involved in the blood coagulation process can therefore impair corresponding encoded proteins and eventually lead to undesired thrombosis within the placental vessels, detachment of placenta and then abortion [3]. In this regard, the most important genetic variations have been identified in *methylenetetrahydrofolate reductase* (*MTHFR*), *factor V Leiden* (*FVL*), *factor II prothrombine* and less importantly *factor VIII* genes, which are included in the thrombophilia panel of RPL in most genetic laboratories around the world. However, the clinical importance of other thrombophilic polymorphisms as well as Human Platelet Antigen-1 (*HPA-1*) rs5918 T > C (C12548T) and fibrinogen-β (*FGB*) rs1800790 G > A is still unclear in the pathogenesis of RPL, especially among Iranian population.

Fibrinogen-β is a substantial element in the coagulation pathway which plays a pivotal role in the regulation of endothelial activity and platelet aggregation [4]. It was described that polymorphism of *FGB* gene promoter was associated with increased level of fibrinogen and therefore enhance platelet dependent coagulation processes that eventually lead to various thromboembolic disorders as well as coronary artery diseases [5,6].

The C12548T (L33P) gene variation of human Platelet Antigen-1 (*HPA-1*) has been frequently reported to be associated with various thromboembolic diseases, as well. HPA-1 is resided on glycoprotein GPIIIa which makes an integrin complex on platelet along with GPIIb and acts as a receptor for fibrinogen and von Willebrand factor and thereby plays a pivotal role in platelet activation and induction of thrombosis [7]. It was described that C12548T polymorphism can trigger platelet activation and thrombosis through two possible mechanisms. The first one is induction of the autoimmune response resulting in platelet dysfunction and undesired thrombosis. The second possible mechanism is the augmentation of the receptor to be more responsive to its ligand, and therefore enhanced platelet activation, leading to thrombosis [8].

There are inconsistent results regarding association of two rs5918 and rs1800790 variants with RPL among various populations. Some of the performed studies have shown significant association of rs5918 and rs1800790 variants with RPL [9,10,11,12,13] while some others could find their association only in combination with other thrombophilia gene polymorphisms [14,15].

The frequency of T and C alleles of rs5918 polymorphisms was calculated as 0.8–1 and 0–0.13, respectively, with higher frequency of C allele among European populations. The frequency of G and A alleles of rs1800790 polymorphism were reported within the ranges of 0.08–0.98 and 0.02–0.28, respectively, with a diverse pattern among various populations around the world.

Since there are contradictory results on the association of rs1800790 and rs5918 variants and risk of RPL among most of the populations, in particular regarding the former variant among Iranian samples, they were selected to be examined separately and in combination with each other [9,10,16].

## 2. Materials and Methods

### 2.1. Patient Selection and DNA Isolation

The present case-control study was performed on 110 cases and 110 controls (19–43 years old) with the same race and ethnicity whom were referred to multicenter women reproductive health offices, Tehran, Iran, during 2016–2017. Diagnostic work up was included hysteroscopy, check for infections, hormonal status, antiphospholipid syndrome, lupus anticoagulant and parental karyotypes.

Inclusion and exclusion criteria were considered according to the defined quality standards which have been previously published [17,18,19]. Included control samples had at least two live births with neither medical nor familial history of abortion and thromboembolic diseases. The case group included every woman with a history of at least two primary consecutive unexplained abortions before 20 weeks of pregnancy. Women with spontaneous abortions, which had been confirmed to have chromosomal abnormalities, maternal anatomic aberrations and hormonal imbalances were excluded from further study. All the enrolled participants filled in the consent form according to the Declaration of Helsinki of 1975 (https://www.wma.net/what-we-do/medical-ethics/declaration-of-helsinki/), revised in 2008 (Approval number of Ethics Committee: ir.tums.rec.1394.553). DNA was extracted using QIAamp DNA Blood Mini Kit (Qiagen, Hilden, Germany) according to the manufacturer’s instructions, from three fresh whole blood samples collected in thylenediaminetetraacetic acid (EDTA) canonical tubes. Isolated DNAs were stored at −20°C until further processing and their quality and quantity were determined using NanoDrop ND-1000 spectrophotometer (Thermo Fishcer Scientific, Waltham, MA, USA).

### 2.2. Polymerase Chain Reaction-Restriction Fragment Length Polymorphism

Two selected polymorphisms were analyzed through polymerase chain reaction-restriction fragment length polymorphism (PCR-RFLP). The primer pairs which were used for amplification of each DNA template, were allele specific and designed through online primer 3 program (Table 1). The PCR mixture included 10 pmol of each forward and reverse primers, 2.5 μL of 10× buffer including 1.5 mM MgCl_2_, 0.2 mM of dNTP mixture and 1 U of Taq DNA polymerase (Qiagen, Hilden, Germany) in addition to 100 ng of each genomic DNA sample adjusted with ddH_2_O up to final volume of 25 μL. After an initial denaturation at 95 °C for 5 min, 30 cycles of PCR was performed according to the following program in a Biorad thermocycler (Bio-Rad Laboratories, Hercules, CA, USA): denaturation at 95 °C for 30 s, annealing temperature specific for each primer pairs for 30 s, extension at 72 °C for 60 s and final extension at 72 °C for 10 min. RFLP reactions were performed on PCR products using Sau96I restriction enzyme for digestion of products related to the variant of *FGB* rs1800790 G > A while the MspI was employed to digest PCR products of *HPA-1* rs5918 T > C polymorphism. PCR and RFLP products were resolved on agarose gel electrophoresis which was then stained with SYBR Green I nucleic acid gel stain (Sigma Aldrich, Castle Hill, NSW, Australia).

### 2.3. Statistical Analysis

By considering the power of study as 95% (α = 0.05), the sample size was determined using Quanto software v1.2 [20]. Fisher’s exact and chi-square tests were used for comparing the frequency of genotypes and alleles of studied polymorphisms between case and control groups. A two-tailed *p*-value < 0.05 was accepted as significant and confidence interval (CI) was considered as 95%.

## 3. Results

Among cases, 87 (79%), 18 (16.3%) and 5 (0.04%) women had two, three and more than three consecutive abortions, respectively (Table 2).

**Figure 1:**
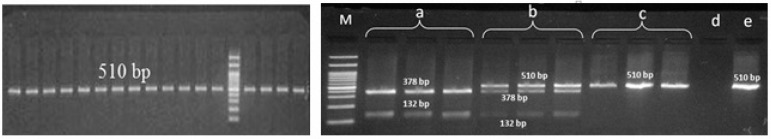
Polymerase chain reaction-restriction fragment length polymorphism (PCR-RFLP) products of *HPA-1* gene variant; M: marker, a: mutant homozygote, b: heterozygote, c: normal homozygote, d: negative control, e: PCR product.

**Figure 2:**
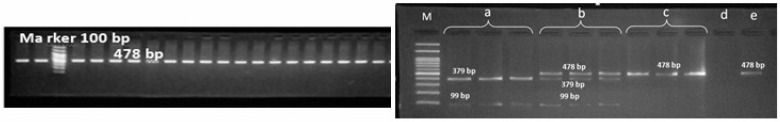
Polymerase chain reaction-restriction fragment length polymorphism (PCR-RFLP) products of *FGB* gene variant; M: marker, a: mutant homozygote, b: heterozygote, c: normal homozygote, d: negative control, e: PCR product.

The genotyping results of *HPA-1* and *FGB* genes variants were presented in Figure 1 and Figure 2, respectively. Considering CI of 95% (0.601–264.140) Pearson chi-square analysis demonstrated meaningful difference in genotype frequency of rs5918 T > C polymorphism of *HPA-1* gene, but not alleles frequency, among cases and controls (*p*-value = 0.02, chi2 = 4.79, odds ratio = 12.6) (Table 3).

Regarding rs1800790 G > A polymorphism of *FGB* gene, no significant association was found between neither genotypes nor alleles and risk of RPL (*p*-value > 0.05) (Table 4).

In addition, the frequency of genotypes and alleles of both studied polymorphisms were in the Hardy–Weinberg Equilibrium among the control group (Table 3 and Table 4). The cumulative effect of both polymorphisms had a significant impact on RPL risk (*p*-value = 0.03).

## 4. Discussion

In the present study, in contrast to *FGB*-455G/A polymorphism, meaningful association of rs5918 T > C polymorphism genotypes of *HPA-1* gene was primarily found with risk of RPL in a subset of Iranian population. To make clearer the role thrombophilia genetic alterations on the mechanism of recurrent abortion, we included the patients with history of 2 ≥ primary abortions and the case and control groups were matched for age and ethnicity. Consistent with our findings, Shi and coworkers, in a meta-analysis of four populations, demonstrated that rs5918 can increase risk of RPL through augmentation of immunological responses and coagulation activity and thereby placental dysfunction [21].

Similar to our results, Maziri P. et al. and Zonouzi et al. could not find any association between rs1800790 G > A polymorphism of *FGB* gene and RPL risk, although they did not identify the primary or secondary RPL status of their case group [16,22]. However, Torabi R. et al. and Bigdeli have found strong difference in frequency of *FGB*-455G/A polymorphism among RPL cases and healthy controls among Iranian samples (allele A frequency among cases: 19.75%/19.5%, respectively) with considerably different sample size [9,10]. However, the obtained positive correlation may be obscured by the main fact that the primary or secondary status of RPL group was not defined in none of two aforementioned studies. In addition, no ethnicity and age matching were considered between two case and control groups in the former study. Li et al. in a meta-analysis of 12 assays which had been performed on the association of *β-fibrinogen-455G/A* polymorphism and risk of RPL among Asian and *Caucasian* populations, no significant difference was found between case and control groups [23]. Al-Astal et al. could not find any association between *FGB*-455G/A polymorphism and RPL risk among Palestinian women—they calculated the frequency of A and G alleles as 17.6% and 82.4% among RPL patients, which is close to our results and could be a further support of our findings [24].

Herein, it was found that combination of two rs5918 and rs1800790 polymorphisms was meaningfully associated with the risk of RPL. Goodman C.S. et al. and Coulam C.B. et al. independently demonstrated meaningful association between rs5918 and rs1800790 variants within a combination panel of nine and ten polymorphisms, respectively, with risk of RPL [14,15]. In contrast, Hohlagschwandtne M. et al. could not find any association between risk of idiopathic recurrent abortion and rs5918 T > C polymorphisms either alone or in combination with other thrombophilia variants among cases with either primary or secondary RPL [25]. Ozdemir O. et al., did not find any association between the risk of RPL with neither rs5918/rs1800790 nor their combination with other thrombophilia genetic mutations in a reliable sample size. However, they did not match case and control group for age and ethnicity and notably considered the cases with early RPL (abortion < 12 weeks of gestation) [26]. That finding may rely on the fact that single nucleotide polymorphism (SNP)–SNP interactions and their effects on abortion pathogenesis may be different in the first and second trimesters of gestation.

In general, discrepancy in the results of various studies even on the same population could be affected by patient selection bias, clinical heterogeneity, different ethnicity of enrolled samples and small sample sizes. In most of the studies, the correlation of abortions number and the primary or secondary type of abortion was not investigated with genetic polymorphisms as well as rs1800790 or rs5918.

This combinatory effect of multiple gene variation on higher risk of RPL can be explained with the possible gene–gene or SNP–SNP interactions, which can critically modulate the risk. On the other hand, those genetic interactions may be affected by lifestyle and environmental changes in different populations [27]. Moreover, *FGB*-455G/A polymorphism may have a role in increasing serum fibrinogen level and subsequent coagulation and thrombosis formation in the pathogenesis of RPL [28]. Although the association of *FGB*-455G/A polymorphism with RPL has been found to be meaningless in most of the studies, a combination of it with other thrombophilic variants was shown to be significant [23]. In addition, -455G/A polymorphism has been demonstrated as a significant gene variant associated with ischemic strokes in various studies [29]. The role of this polymorphism in pathogenesis of RPL, therefore, should be investigated in more populations and studies. Moreover, investigation of the effect of gestational week of abortion (first or second trimester) on its association with thrombophilia genetic variation is the other important factor which can be included in sample selection of future studies.

Finally, genetic analysis for hereditary thrombohilia has been suggested to be done for only women with additional thrombophilia risk factors according to the recent ESHRE guidelines on RPL, consideration of *FGB*-455G/A and *HPA-1* rs5918 T > C polymorphisms in thrombophilia panel of RPL patients may improve diagnosis rate of those patients (https://www.eshre.eu/Guidelines-and-Legal/Guidelines/Recurrent-pregnancy-loss). Further studies on larger sample sizes, considering additional factors including gestational week of abortion, primary and secondary RPL group, control group with or without history of abortion, lifestyle and environmental elements, are required to show the significance of the two studied gene polymorphisms in determining the risk of RPL.

## 5. Conclusions

Herein, the association of rs5918 T > C polymorphism of *HPA-1* gene was initially found among the Iranian population affected by RPL. Although the literature on the single association of *FGB*-455G/A polymorphism with RPL seems to be poor, most of them did not find significant results. However, a combination of it with other thrombophilia variants was almost meaningfully associated with increased risk of recurrent abortion. According to the previous findings and functional correlation between human platelets antigens and fibrinogens chains including beta, the inclusion of 455G/A polymorphism in the thrombophilia panel may enhance diagnosis and treatment of women with thrombophilia disorders. Of note, association of no one of the other genetic variants of thrombophilia panel which is currently carried out around the world has been universally approved in all of the performed studies. Further studies considering larger sample sizes are warranted to clinically and statistically determine the critical inclusion of rs5918 T > C and especially 455G/A polymorphisms in diagnostic laboratory panels.

## Figures and Tables

**Table 1:** Primer pairs sequences used to amplify fibrinogen β chain (*FGB)* and human platelets antigen 1 (*HPA-1)* polymorphisms.

Gene (Polymorphism)	Primer Sequence (5′–3′)	T (°C) *
*FGB* rs1800790 G > A	F: CATTTAGTCTGTGAGCATAC′ R: ACCTACTCACAAGGCAACCA	52
*HPA-1* rs5918 T > C	F: CCTTCTAGCTACAACTCCATGA R: GGGACTGACTTGAGTGACCTG	60

* Ta: Temperature of annealing.

**Table 2:** Characteristics of case and control groups.

	Cases	Controls
Age	33.13 ± 6.2	32 ± 6.36
Number of abortion	2.28 ± 0.67	0
Number of live birth	0	2.5 ± 1.02

**Table 3:** Frequency of genotypes and alleles of rs5918 T > C polymorphism of *HPA-1* gene.

	Controls (%)	Cases (%)	*p* Value	HWE *
**Alleles**				
T	202 (91.81%)	198 (90%)		
C	18 (8.18%)	22 (10%)		
Total	220 (100)	220 (100)	0.08	
**Genotypes**				
TT	95(86.36%)	88 (80%)		
TC	12 (10.91%)	22 (20%)		
CC	3 (2.73%)	0 (0%)		
Total	110 (110)	110 (100)	0.02	0.24

* Abbreviation: HWE: Hardy–Weinberg Equilibrium *p*-value (Pearson).

**Table 4:** Frequency of genotypes and alleles of rs1800790 G > A polymorphism of *FGB* gene.

	Controls (%)	Cases (%)	*p* Value	HWE *
**Alleles**				
G	27 (12.3%)	199 (90.45%)		
A	193 (87.7%)	21 (9.55%)		
Total	220 (100)	220 (100)	>0.05	
**Genotypes**				
GG	0 (0%)	91 (82.73%)		
GA	27 (24.55%)	17 (15.45%)		
AA	83 (75.45%)	2 (1.82%)		
Total	110 (100)	110 (100)	>0.05	0.14

* Abbreviation: HWE: Hardy–Weinberg Equilibrium *p*-value (Pearson).
